# Targeting the DNA Double Strand Break Repair Machinery in Prostate Cancer

**DOI:** 10.1371/journal.pone.0020311

**Published:** 2011-05-23

**Authors:** Fadhel S. Shaheen, Pawel Znojek, Ann Fisher, Martin Webster, Ruth Plummer, Luke Gaughan, Graeme C. M. Smith, Hing Y. Leung, Nicola J. Curtin, Craig N. Robson

**Affiliations:** 1 The Northern Institute for Cancer Research, Medical School, Newcastle University, Newcastle Upon Tyne, United Kingdom; 2 The Beatson Institute for Cancer Research, Glasgow, United Kingdom; 3 KuDOS Pharmaceuticals Ltd, Cambridge, United Kingdom; University Medical Center Hamburg-Eppendorf, Germany

## Abstract

Regardless of the achievable remissions with first line hormone therapy in patients with prostate cancer (CaP), the disease escapes the hormone dependent stage to a more aggressive status where chemotherapy is the only effective treatment and no treatment is curative. This makes it very important to identify new targets that can improve the outcome of treatment. ATM and DNA-PK are the two kinases responsible for signalling and repairing double strand breaks (DSB). Thus, both kinases are pertinent targets in CaP treatment to enhance the activity of the numerous DNA DSB inducing agents used in CaP treatment such as ionizing radiation (IR). Colony formation assay was used to assess the sensitivity of hormone dependent, p53 wt (LNCaP) and hormone independent p53 mutant (PC3) CaP cell lines to the cytotoxic effect of IR and Doxorubicin in the presence or absence of Ku55933 and NU7441 which are small molecule inhibitors of ATM and DNA-PK, respectively. Flow cytometry based methods were used to assess the effect of the two inhibitors on cell cycle, apoptosis and H2AX foci formation. Neutral comet assay was used to assess the induction of DNA DSBs. Ku55933 or NU7441 alone increased the sensitivity of CaP cell lines to the DNA damaging agents, however combining both inhibitors together resulted in further enhancement of sensitivity. The cell cycle profile of both cell lines was altered with increased cell death, DNA DSBs and H2AX foci formation. This study justifies further evaluation of the ATM and DNA-PK inhibitors for clinical application in CaP patients. Additionally, the augmented effect resulting from combining both inhibitors may have a significant implication for the treatment of CaP patients who have a defect in one of the two DSB repair pathways.

## Introduction

According to the U.S National Institutes of Health, the age-adjusted incidence rate of prostate cancer 2003–2007 was 156.9 per 100.000 men per year. Although high response rates can be achieved by first line therapy with surgery, radiotherapy, antiandrogen or their combinations; the natural progress of the disease is towards the hormone refractory status [1] where chemotherapy is the most effective treatment but still not curative [2]. This resistance highlights the importance of identifying new targets that can increase the sensitivity of CaP cells and hence the response rates and overall survival of patients. Ataxia telangiectasia mutated (ATM) and the DNA dependent protein kinase catalytic subunit (DNA-PKcs) are members of the phosphatidyl inositol 3-kinase related kinases (PIKK) superfamily. Members of this family are characterised by their high molecular weight and sequence similarity to the p110 subunit lipid kinase PI3-kinase [3]. In mammalian cells, ATM and DNA-PK play key roles in the DNA double strand break (DSB) response, via homologous recombination (HR) and non homologous end joining (NHEJ), respectively [4], [5]. Rapid phosphorylation of both ATM and DNA-PK occurs in response to DSB following endogenous or exogenous insults. Once activated, ATM and DNA-PK signal to a wide spectrum of downstream targets that are involved in the repair process, cell cycle regulation and apoptosis [6]. The choice of which pathway repairs the DSB is cell cycle stage dependent, with NHEJ being the dominant pathway in G0 and G1, and HR dominates in S and G2/M phases [7]. ATM and DNA-PK are cleaved by caspase 3 once the decision to activate apoptosis is made in the cell and this cleavage event is thought to facilitate apoptosis by disabling the DNA signalling and repair machinery [8], [9]. Traditional PI3K inhibitor, wortmannin with generally low selectivity against different classes and/or isoforms of PIKK has been widely used to study ATM and DNA-PK signalling pathways [10]. Ku55933 was identified as a potent and specific ATP competitive inhibitor of ATM (IC_50_ 13 nmol/L) with respect to the inhibition of other members of the PIKK family. Ku55933 increased the sensitivity of breast cancer cells to IR, altered their cell cycle profile, and inhibited the phosphorylation of a panel of ATM targets. A–T cells did not show these effects when treated with Ku55933 [11]. NU7441 was identified as a potent and specific ATP competitive inhibitor of DNA-PK (IC_50_ 14 nmol/L) with 100-fold selectivity for DNA-PK relative to other members of the PI3KK family. NU7441 increased the sensitivity of colon cancer cells to IR and topoisomerase II inhibitors, and altered their cell cycle profile. DNA-PK deficient V3 cells did not show these effects when treated with NU7441 [12]. This study was designed as a preclinical evaluation of both ATM and DNA-PK inhibitors to investigate whether these inhibitors can improve the efficacy of DNA DSB inducing agents for CaP treatment.

## Materials and Methods

### Chemicals

All routine chemicals were purchase from Sigma unless otherwise stated. NU7441 was synthesised at the Northern Institute for Cancer Research, Newcastle University (Newcastle upon Tyne, UK) in collaboration with KuDOS Pharmaceuticals (Cambridge, UK). KU-55933 was a kind gift from KuDOS Pharmaceuticals. Both inhibitors were dissolved in DMSO at a stock concentration of 2000× the final concentration required for the experiment. Doxorubicin was dissolved in water at stock concentration of 1000× the final concentration required for the experiment. All compounds were stored in aliquots at −20°C.

### Cell culture

LNCaP hormone sensitive (p53 wild type) and PC3 hormone insensitive (p53 null) CaP cell lines were purchased from ATCC and cultured in RPMI 1640 medium supplemented with 10% (v/v) FCS. Both cell lines were routinely screened for Mycoplasma.

### Colony formation assay

Cells were seeded in 6 well plates at different densities. After allowing to attach, cells were incubated with Ku55933 (10 µM) alone or in combination with NU7441 (1 µM) for 1 h before either irradiation (0–2 Gy) or doxorubicin treatment (0–75 nM). 24 h later, wells were gently washed twice with warm PBS before adding fresh medium and returned to the incubator for 7–10 days for PC3 cells and 12–14 days for LNCaP cells to form colonies. Colonies were fixed with Carnoy fixative, dried, stained with 0.4% crystal violet and counted manually. The survival reduction factor was calculated as the surviving fraction of cells in the absence of the inhibitors divided by the surviving fraction of cells in the presence of inhibitors for any given dose or concentration of cytotoxic agent.

### Flow cytometry based method for cell cycle

Cells were seeded in 6 well plates. 24 h later, cells were treated with 1 µM NU7441 and/or 10 µM Ku55933 prior to IR or doxorubicin treatment. 48 h later, cells were collected in FACS tubes (Becton & Dickinson, Oxford UK) maintaining the growth medium before being stained with propidium iodide (PI). Samples were analyzed directly on a FACScan (Becton & Dickinson), fluorescence intensity in arbitrary units was plotted in histograms, and the cell cycle phases were determined using WINMDI version 2.8 (The Scrrips Research Institute, USA).

### Flow cytometry based method for active caspase 3

Cells were seeded in 6 well plates and treated with the inhibitors and/or DNA damaging agent as previously described and collected in FACS tubes maintaining the growth medium. Cells were stained with FITC conjugated active caspase 3 antibody according to the manufacturer's instructions (Becton Dickinson, Oxford UK) and analyzed directly on a FACScan. Fluorescence intensity in arbitrary units was plotted in dot plots, and the mean fluorescence intensity was calculated using WINMDI version2.8.

### Flow cytometry based method for γ-H2AX foci formation

Cells were seeded in 6 well plates, treated with the inhibitors and/or DNA damaging agent as previously described and collected in FACS tubes. Cells were stained using FITC conjugated γH2AX antibody as described previously [13]. Samples were analyzed directly on a FACScan. Fluorescence intensity in arbitrary units was plotted in histograms, and the mean fluorescence intensity was calculated using WINMDI version2.8.

### Western blotting

Whole cell extracts were prepared 6 h after 1 µM doxorubicin and 1 h after 10 Gy IR in the presence and absence of the inhibitors. Equivalent amounts of cell lysate were loaded onto 4.5, 6, 12 and 15% gels alongside a protein marker SeeBlue® Pre-Stained Standard (Invitrogen, USA). Gels were electrophoresed and proteins were transferred to Hybond-C membrane. ECL-system (GE Healthcare, Buckinghamshire, UK) was used to detect antibody conjugated proteins according to the manufacturer's instructions and visualised by exposure to X-ray film (Kodak, Herts, UK). Anti DNA-PK, anti ATM, anti DNA-PK _Ser2056_ and anti DNA-PK_Thr2906_ were purchased from (Abcam, Cambridge UK). Anti ATM_Ser1981_, anti H2AX_Ser139_ and anti p53_Ser15_ were purchased from (Gene Tex, USA), (Upstate, UK) and (Cell signalling, UK), respectively.

### Neutral comet assay

PC3 were subjected to the neutral comet assay according to the manufacturer instructions (Trevigen, Inc., Gaithersburg, MD, USA). Two main modifications were applied, the cells were lysed for 1 h and electrophoresed for 30 minutes at 30 V. Comets were visualized by fluorescence Leica DMR microscope and analyzed using Komet 5.5 (kinetic imaging Ltd, Nottingham, UK).

### Statistical analysis

Statistical analysis was applied using prism software version4 (GraphPad Prism, San Diego, USA).

## Results

### Ku55933 and NU7441 inhibit ATM and DNA-PK kinase activity respectively in IR and doxorubicin treated CaP cells

**Figure 1:**
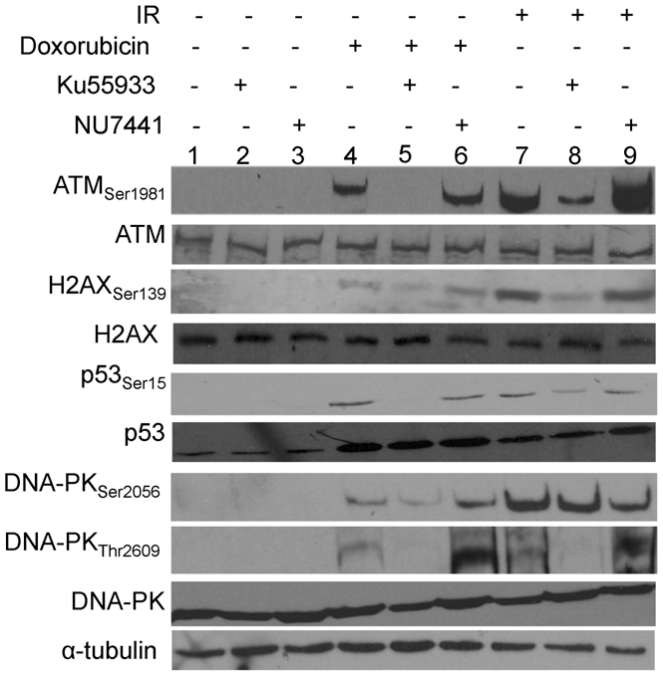
Ku55933 and NU7441 inhibit ATM and DNA-PK, respectively. Whole LNCaP cell extracts were prepared 1 h after 10 Gy and 6 h after 1 µM doxorubicin ±10 µM Ku55933 or 1 µM NU7441. Extracts were analyzed by western blotting and probed with the indicated antibodies. Data is a representative of three independent experiments.

ATM-dependent ATM_Ser1981_, p53_Ser15,_ H2AX_Ser139_ and DNA-PK_Thr2609_ phosphorylation were induced 1 h after 10 Gy and 6 h after 1 µM doxorubicin and were inhibited by Ku55933. IR induced DNA-PK_Ser2056_ phosphorylation (reported as DNA-PK autophosphorylation site) was inhibited by NU7441 (as expected) and surprisingly doxorubicin induced DNA-PK_Ser2056_ phosphorylation was also inhibited by Ku55933. NU7441 did not inhibit ATM_Ser1981_, p53_Ser15,_ H2AX_Ser139_ and DNA-PK_Thr2609_ phosphorylation (Figure 1).

### Ku55933 and NU7441 are minimally cytotoxic to CaP cells

**Figure 2:**
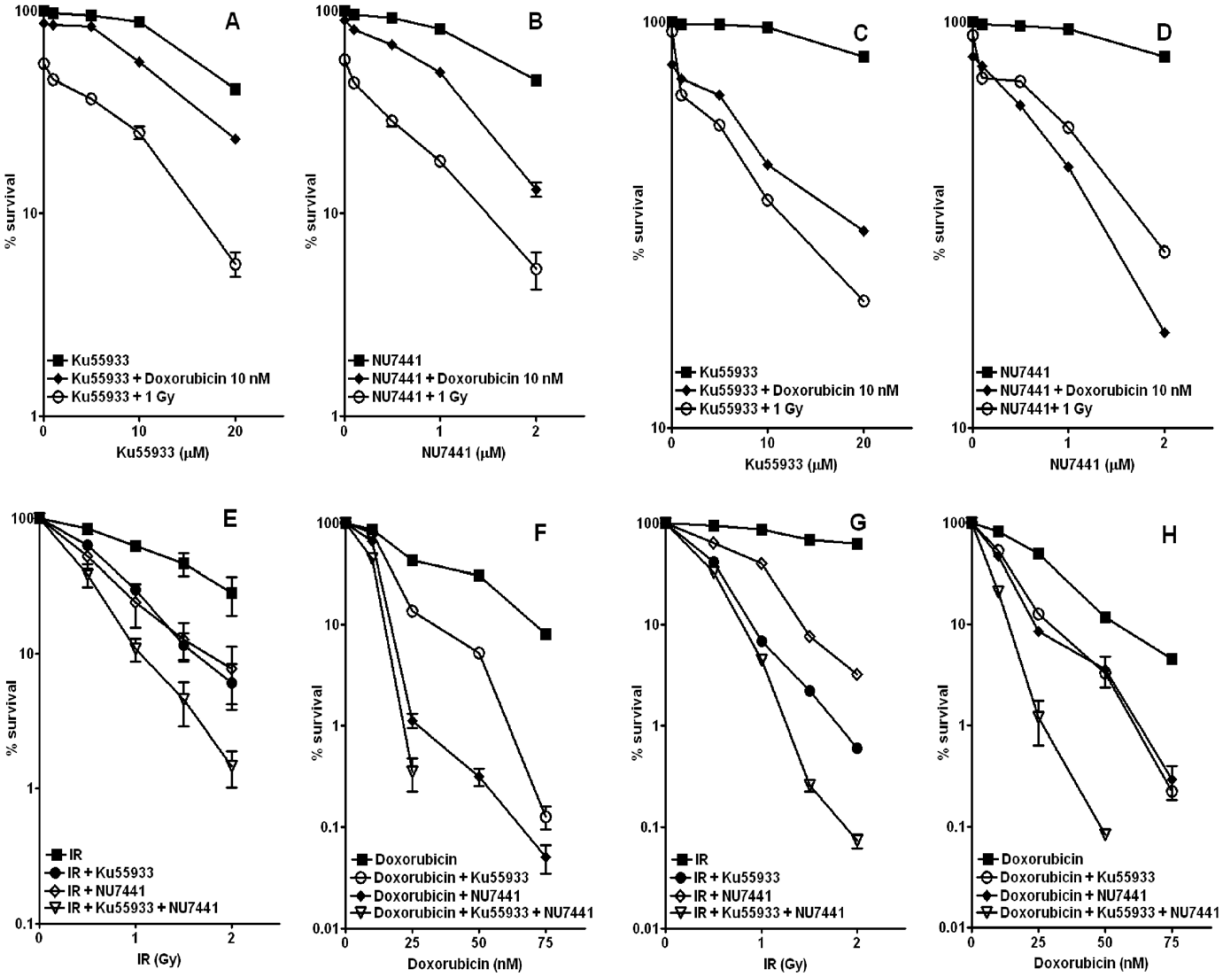
Ku55933 and/or NU7441 sensitised CaP cells to IR or doxorubicin. **LNCaP (A&B)** or **PC3 (C&D)** cells were seeded in different densities and left to attach before 1 h incubation with varying concentration of Ku55933 or NU7441 prior to irradiation 1 Gy or 10 nM doxorubicin treatment for 24 h (*P*<0.001). **LNCaP (E&F)** or **PC3 (G&H)** cells were seeded in different densities and left to attach before 1 h incubation with Ku55933 (10 µM) or NU7441 (1 µM) prior to treatment with varying IR doses or doxorubicin concentration for 24 h (*P*<0.001). All results are the mean of three independent experiments ± SEM.

In order to determine the intrinsic cytotoxicity of Ku55933 and NU7441 in relation to chemo and radiosensitization, cells were exposed to increasing concentrations of Ku55933 or NU7441 with minimally cytotoxic concentration of IR (1 Gy) or doxorubicin (10 nM). Both Ku55933 (10 µM) and NU7441 (1 µM) caused minimal or no cytotoxicity in LNCaP (Figure 2A&B) and PC3 (Figure 2C&D). Each inhibitor significantly increased IR and doxorubicin cytotoxicity in both CaP cell lines in an inhibitor dose-dependent manner. These two concentrations (10 µM Ku55933 or 1 µM NU7441) were used in subsequent experiments.

### Ku55933 and NU7441 reduced CaP cell lines cellular survival in response to IR or doxorubicin

Having demonstrated a concentration-dependent chemo and radiosensitization in both cell lines, we then investigated the response to a fixed concentration of each inhibitor with increased concentrations of doxorubicin or IR dose. There was a dose dependent decrease in cell survival in response to IR. PC3 cells displayed resistance to IR compared to LNCaP, combining NU7441 or Ku55933 with IR significantly reduced the cell survival for LNCaP (Figure 2E) and substantially for PC3 (Figure 2G). Similarly, doxorubicin reduced cell survival in both cell lines, Further chemo-sensitization resulted from combining NU7441 or Ku55933 in LNCaP (Figure 2F) and PC3 (Figure 2H). Combining both inhibitors significantly increased the chemo-radiosensitization and NU7441 combination was more effective in inducing chemosensitisation in LNCaP whereas Ku55933 combination had more effect in inducing radiosensitization in PC3 suggesting an advanced role for ATM in the hormone refractory model.

### Ku55933 and NU7441 altered cell cycle and increased their apoptosis in response to DNA damage in CaP cell lines

**Figure 3:**
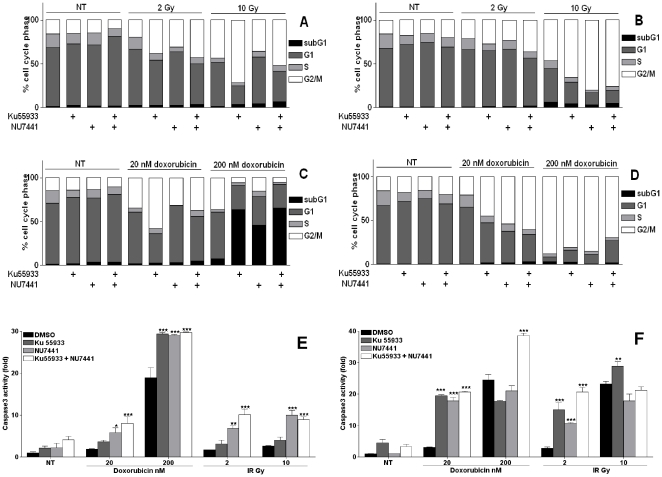
Ku55933 and NU7441 altered CaP cell lines cell cycle and increased apoptosis. **LNCaP (A&C)** and **PC3 (B&D)** cells were incubated with Ku55933 (10 µM) and/or NU7441 (1 µM) before being exposed to the indicated doses of IR or doxorubicin for 48 h and then stained with PI and analysed directly on FACscan. Data is a representative three independent experiments. **LNCaP (E)** and **PC3 (F)** cells were treated as described above and stained with FITC-conjugated anti caspase 3 antibody before direct analysis on FACScan. Results are the mean of three independent experiments ± SD.

We next determined whether the increased sensitivity of CaP cell lines is accompanied by increased apoptosis or changes in the cell cycle profile. IR caused minimal/no increase in LNCaP (Figure 3A) compared to a dose-dependent increase in G2 accumulation in PC3 (Figure 3B). Additionally, the combination of both inhibitors had an additive effect on the G2 accumulation at low dose (2 Gy) but not at high dose (10 Gy). Doxorubicin treatment caused cells to accumulate in G2/M especially at high dose. In general, combining either inhibitor with a low dose doxorubicin (20 nM) increased G2/M accumulation although NU7441 had only modest effect in LNCaP cells (Figure 3C). Whereas the single inhibitor combination at high dose doxorubicin (200 nM) resulted in a striking increase in subG1 in LNCaP (Figure 3C) compared to an interesting increase in G1 in PC3 cells (Figure 3D). An additive effect was observed when combining both inhibitors.

IR induced a dose dependent cell death in both cell lines. LNCaP apoptosis was enhanced when combining Ku55933 and/or NU7441 (Figure 3E). However, PC3 cell death (Figure 3F) was increased in response to the inhibitor combinations at low dose only. The increased apoptosis caused by the combination of both inhibitors is consistent with the survival data showing radio-sensitisation was more pronounced for both cell lines treated with both inhibitors together. Additionally, Ku55933 induced greater cell death in PC3 cells compared to NU7441 which was a consistently observed effect of Ku55933 on the survival of irradiated PC3 cells. Doxorubicin caused a dose-dependent increase in caspase 3 activity in both cell lines. Combining Ku55933 and/or NU7441 with 20 nM doxorubicin enhanced the cell death of LNCaP (Figure 3E) and PC3 (Figure 3F) cells. A massive increase in LNCaP apoptosis resulted from Ku55933 and/or NU7441 combined with 200 nM doxorubicin, 95–99% of cells were positive for active caspase3, whereas no increase was detected in PC3 under the same conditions.

Finally, the apoptosis data is consistent with survival data as the massive induction of apoptosis correlates with severe reduction in the cellular survival at high doses of doxorubicin. Furthermore, NU7441 was superior to the Ku55933 in inducing apoptosis in LNCaP cells. Whereas, Ku55933 had greater effect in PC3 cells which is consistent with the survival data.

### Ku55933 and NU7441 increase the induction of DNA DSB in CaP cell lines

**Figure 4:**
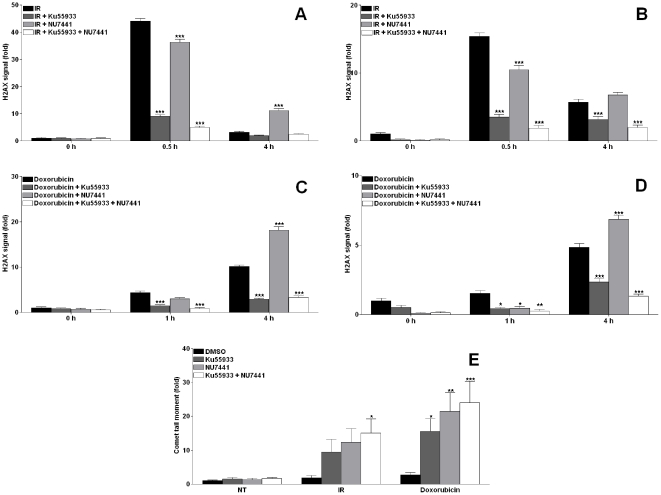
Ku55933 and NU7441 increased DNA DSB in CaP cell lines. **LNCaP (A&C)** and **PC3 (B&D)** cells were treated with 10 Gy or 1 µM doxorubicin following 1 h incubation with Ku55933 (10 µM) and/or NU7441 (1 µM) and stained with FITC-conjugated H2AX antibody before being analysed directly on FACscan. Results are the mean of at least 5 independent experiments±SEM. **PC3 (E)** cells were treated as above for 24 h and analysed using comet assay, 50 cells were counted per arm treatment. Results are the mean of 3 independent experiments ± SEM.

We investigated whether the increase in CaP cell lines sensitivity in response to ATM and/or DNA-PK inhibition is a result of increased number of DNA DSBs by a direct measure of H2AX signal and a direct measure of DNA DSB (neutral comet assay). As anticipated γ-H2AX signal increased 0.5 h following IR in both cell lines and returned to normal levels within 4 h in LNCaP cells (Figure 4A) whereas it was only reduced by 50% in PC3 cells (Figure 4B) which suggest greater repair capacity in LNCaP compared to PC3. Doxorubicin treatment caused time-dependent increase in γ-H2AX in LNCaP (Figure 4C) and PC3 (Figure 4D). Combining either Ku55933 or both inhibitors with either IR or doxorubicin reduced the γ-H2AX signal in both cell lines at all time points examined which is consistent with ATM being the major kinase responsible for H2AX phosphorylation and the inability of DNA-PK to phosphorylate H2AX in the presence of inhibited ATM. NU7441 modestly inhibited the initial γ-H2AX induction in both cell lines following IR and doxorubicin. However, at later time points inhibiting DNA-PK resulted in increased γ-H2AX signal reflecting increased DNA DSBs highlighted by γ-H2AX, a consequence of phosphorylation by the active ATM. Noteworthy, in experiments where both kinases were inhibited there was no further increase in H2AX signal. PC3 cells showed a small increase in the comet tail moment 24 h following IR or doxorubicin reflecting their high capability to repair their DNA damage lesions. However, combining either inhibitor with either DNA damaging agent caused modest increase in the tail moment. Combining both inhibitors had an additive increase in the tail moment (Figure 4E).

Noteworthy, the increase resulting from the doxorubicin treatment was higher compared to that resulting from IR; this could be in part related to the fact that IR mainly induces higher level of single strand breaks.

## Discussion

Ku55933 and NU7441 are potent inhibitors of ATM and DNA-PK, respectively. Each inhibitor abolished the autophosphorylation of its own protein target in both CaP cell lines following IR. The data with doxorubicin treatment was similar between the two cell lines but the phosphorylation of DNA-PK_Ser2056_ appears not to be inhibited by NU7441. Notably, the phosphorylation of the downstream targets H2AX_Ser139_ and p53_Ser15_ which are mainly regulated by ATM was abolished when both inhibitors were combined. Furthermore, a novel phosphorylation event is reported here as the phosphorylation of Ser2056 of DNA-PKcs, believed to be a true autophosphorylation site for DNA-PK [14], was reduced following Ku55933 treatment (not NU7441) in response to doxorubicin but not IR treatment suggesting this site could be targeted by ATM, depending on the DNA damage inducing agent. A relevant control would be to deplete native ATM and substitute it with a kinase-dead ATM.

Combining either inhibitor with IR or doxorubicin resulted in a massive and significant increase in the sensitivity of LNCaP and PC3 cells to the cytotoxic effect of both IR and doxorubicin. Additionally, combining the two inhibitors together with DNA damaging agents resulted in further enhancement in the cytotoxicity compared to single inhibitor combination. This concurs with reported literature where targeting ATM and DNA-PK using siRNA methodology revealed increased sensitivity to IR and to chemotherapy in different CaP cell lines [15]. The increased sensitivity resulting from the combination of both inhibitors could have a significant implication for the treatment of CaP in patients with a defect in the HR or NHEJ pathways such as BRCA mutation carriers [16]. Patients with defect in one repair pathway could potentially be treated with the inhibitor of the other pathway and benefit from maximum treatment outcome with minimal toxicity.

Herein, a major characteristic of the PC3 cell cycle, lack of p53 function, led to an increased accumulation in the G2/M phase of the cell cycle in the presence of ATM, DNA-PK or both inhibitors particularly when combined with IR treatment. However, inhibiting either ATM or DNA-PK or both in combination with high dose doxorubicin treatment increased the G1-phase accumulation, suggesting activation of the G1 checkpoint by other pathways such as the p38MAPK/MK2 [17]. In the presence of functional p53 (LNCaP cells), cells accumulated in G2/M-phase when ATM was inhibited while they accumulated in G1 when DNA-PK was inhibited. This could explain the survival data since the accumulation in G2 would allow more NHEJ mediated DNA repair. This data supports previous reports suggesting that the effect of DNA-PK on p53 contributes to the apoptosis process rather than to cell cycle control. It is interesting that ATM inhibition leads to G2 accumulation which may be explained by the effect of kinase active ATR in the absence of active ATM kinase [18]. Interestingly, combining either inhibitor or the dual inhibitor combination with high dose doxorubicin (excessive DNA damage) resulted in a dramatic increase in the subG1 population, an indication of apoptosis. This suggests that the presence of p53 can trigger cell death when the damage in DNA is too substantive to be repaired. Our data is consistent with a role for ATM and DNA-PK in cell cycle regulation through their effect on different proteins [19].

In this study, the effect of inhibiting ATM or DNA-PK on the cellular death of CaP cell lines revealed that, independent of p53 status or the hormone dependency, this inhibition increased the programmed cell death induced by low doses of either doxorubicin or IR. However, the presence of p53 (LNCaP cells) greatly increased the apoptotic population with high dose doxorubicin. Whereas, in the absence of p53 (PC3 cells) there was either none or a small increase in cell death with high dose doxorubicin which is consistent with observed increase in cell cycle arrest. The more pronounced apoptosis in PC3 compared to LNCaP at low doses of DNA damage could be explained by the shorter period of treatment (48 h) for LNCaP compared to (96 h) for PC3 cells.

We observed that the primary induction of H2AX foci was substantially reduced by combining either or both inhibitors with DNA damaging agents. At later time points, H2AX foci formation were still inhibited in the ATM inhibitor combination arms, whereas it increased in the DNA-PK inhibitor arms. This implies that DNA-PK inhibition increases the DNA DSBs that are highlighted by increased number of H2AX foci formed at the damage site. However, decreased H2AX foci formation in the ATM inhibitor arms is not a consequence of less DSBs being generated as we demonstrated increased DNA DSBs using the neutral comet assay. This is consistent with the data suggesting that ATM is the major kinase that induces H2AX foci and that DNA-PK can phosphorylate H2AX only in the absence of ATM but not in the presence of chemically inhibited ATM [20]. This data is consistent with a report using other methods for H2AX detection [21] where the initial H2AX foci formation (30 min following IR) was abolished when combining ATM and/or DNA-PK inhibitors. Another report contradicts with our data [22] where they demonstrated DNA-PK plays dominant role in H2AX foci formation in the absence of ATM function; however this report used cell lines that lack either ATM or DNA-PK which is not the case in our cell lines. Additionally, inhibiting both ATM and DNA-PK did not result in increased H2AX signal at all time points. This suggests that ATR (the third kinase implicated in H2AX phosphorylation) is unable to carry out this phosphorylation function in the presence of ATM and DNA-PK even when both are inhibited. An interesting approach would be to investigate the effect on Rad51 foci formation when these kinases are inhibited.

The key role of ATM and DNA-PK in repairing DNA DSB in mammalian cells suggests that knocking down the function of either or both proteins should increase the numbers of DNA DSBs following an inducing treatment. Using the neutral comet assay we showed that inhibition of ATM and/or DNA-PK resulted in increased number of DNA DSBs following IR or doxorubicin treatment. An additive effect was observed in the treatment arms where both kinases were inhibited. It is interesting that the comet tails are almost equal in the single inhibitor arms although it would be expected that the DNA-PK inhibitor should cause more increase in DSBs as NHEJ is the major pathway for repairing DNA DSBs. This may be because we conducted the comet assay at 24h, which is more physiologically meaningful to the cells but does not necessarily reflect the repair kinetics. Most DSBs are repaired rapidly by NHEJ and then slower kinetics by HR for residual frank DSBs and DSBs resulting from stalled replication fork. ATM impacts on HR therefore KU55933 should diminish this slow phase even further and therefore a greater number of breaks at 24 hr compared to when HR is not inhibited (control and DNA-PK inhibitor arms).

In summary, this study reports a novel treatment for prostate cancer by targeting the DNA DSBs repair machinery. We show that targeting DSB repair confers an enhanced chemo- and radio-sensitivity in hormone sensitive and insensitive CaP cell lines associated with increased programmed cell death. Further evaluation of the specific inhibitors needs to be conducted in animal models, including the assessment of plasma availability, toxicity, and efficacy before moving further towards any clinical trial.
